# Behavioral changes of metritic primiparous cows treated with chitosan microparticles or ceftiofur

**DOI:** 10.3168/jdsc.2022-0221

**Published:** 2022-06-07

**Authors:** Jessica G. Prim, Eduardo B. de Oliveira, Anderson Veronese, Ricardo C. Chebel, Klibs N. Galvão

**Affiliations:** 1 Department of Large Animal Clinical Sciences, University of Florida, Gainesville 32608; 2 Department of Animal Sciences, University of Florida, Gainesville 32608; 3 D. H. Barron Reproductive and Perinatal Biology Research Program, University of Florida, Gainesville 32608

## Abstract

The main objective was to characterize behavioral changes in metritic primiparous cows treated with chitosan microparticles (CM) or ceftiofur (CEF). A secondary objective was to compare behavioral patterns of metritic cows with nonmetritic (NMET) cows. Nulliparous Holstein cows (n = 311) were fitted with a neck-mounted automated health-monitoring device (AHMD) from −21 to 60 d relative to calving. Cows diagnosed with metritis (d 0), characterized by watery, fetid, red-brownish uterine discharge within 21 d in milk were assigned randomly to CM (n = 45), intrauterine infusion of 24 g of CM dissolved in 40 mL of sterile distilled water on d 0, 2, and 4; CEF (n = 47), subcutaneous injection of 6.6 mg/kg ceftiofur crystalline-free acid on d 0 and 3; and control (CON; n = 39), no treatment. For comparison, NMET cows (n = 180) were matched with metritic cows according to age at calving and calving date. Postdiagnosis, there was an effect of treatment and an interaction between treatment and time on rumination and activity. The interaction showed that CM had lesser rumination than CEF from d 1 to 11, d 18, and d 20; CM had lesser rumination than CON from d 2 to 8; and CEF was not different from CON. The interaction showed that CM had lesser activity than CON on d 2, from d 6 to 11, and d 13 to 14; CM was not different from CEF; and CEF had lesser activity than CON on d 8, 9, 13, and 14. Prediagnosis, cows in CM, CEF, and CON had lesser rumination and activity than cows in NMET. Postdiagnosis, cows in CM, CEF, and CON had lesser rumination than NMET from d 0 to 2 and had lesser activity than NMET from d 0 to 5. In summary, CM decreased rumination and activity compared with CON, which indicates a negative systemic effect of CM. This may be associated with exacerbated inflammation in the uterus. Additionally, metritic cows had decreased rumination and activity prediagnosis, which may allow for the use of AHMD for metritis diagnosis.

## Highlights

• Chitosan microparticles negatively affected the rumination and activity of cows with metritis.
• The negative effect of CM on rumination and activity indicates a negative systemic effect that may be associated with increased inflammation in the uterus.
• Regardless of treatment, cows with metritis had decreased rumination and activity starting 5 days before diagnosis until at least 2 days after diagnosis.
• The automated health-monitoring device was a useful tool to evaluate rumination and activity patterns after metritis treatment.

## Graphical Abstract

**Figure:**
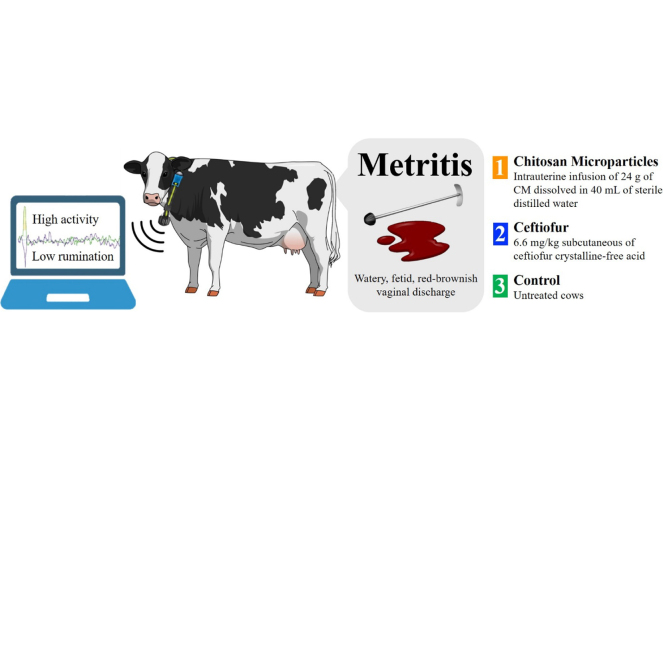
**Summary:** Metritis is a prevalent disease in dairy cows that impairs welfare, performance, and profitability. We used automated health-monitoring devices to compare activity and rumination in lactating primiparous dairy cows with metritis following treatment. Cows treated with chitosan microparticles (CM) had lower rumination and activity than cows treated with ceftiofur and untreated controls. Decreased rumination and activity indicate a negative systemic effect of CM that may be associated with increased inflammation in the uterus.

Behavior patterns can be used as an indicator of animal health because sick cows have decreased feeding and rumination compared with healthy cows (Urton et al., 2005; Huzzey et al., 2007). Observing animal behavior, however, is a labor-intensive and difficult task, especially in large herds. One possible solution is the use of automated health-monitoring devices (**AHMD**), which have been considered an alternative to identification of sick animals (Rutten et al., 2013; Stangaferro et al., 2016). The data collected by AHMD have also been evaluated for their usefulness to predict health disorders (Soriani et al., 2012; Merenda et al., 2021b) and to assist in treatment decisions (Rutten et al., 2013).

Metritis is a common disease that affects dairy cows in the early lactation period. It is characterized by an abnormally enlarged uterus with a watery, fetid, red-brownish uterine discharge (McLaughlin et al., 2012). Metritis causes economic losses mostly because of a decrease in milk yield and reproductive performance, treatment costs, and survival in the herd (Pérez-Báez et al., 2021).

The treatment of metritis generally includes antimicrobial administration, which has been shown to reduce the losses in milk yield, reproductive performance, and profitability (Silva et al., 2021). In the United States, ceftiofur is the antimicrobial of choice because it is labeled for the treatment of metritis and it does not require milk withdrawal (McLaughlin et al., 2012); however, other antimicrobials have been shown to be effective in the treatment of metritis (Lima et al., 2014; Merenda et al., 2021a). Nonetheless, there is increased concern about the contribution of the use of antimicrobials in food animals to the rise in antimicrobial resistance (Van Boeckel et al., 2015; Ma et al., 2021). It is estimated that most of the antimicrobial consumed annually is related to veterinary field and a significant fraction of these antimicrobials are important for human medicine (Van Boeckel et al., 2015). This emphasizes the need for the development of alternatives to traditional blanket antimicrobial treatment of cows diagnosed with metritis.

A derivate of chitin, chitosan is a biopolymer part of the exoskeleton of arthropods and cell walls of fungi and yeast that has broad-spectrum antimicrobial activity (Jeon et al., 2014). de Oliveira et al. (2020) investigated the use of chitosan microparticles (**CM**) for the treatment of metritis and observed detrimental effects on milk yield, fertility, and survival when compared with ceftiofur-treated or no treatment. The decrease in survival was mostly a consequence of increased culling due to failure to cure metritis, pelvic inflammation, peritonitis, and mass in the pelvis, suggesting that CM resulted in exacerbated inflammation in the uterus.

Based on the negative effects observed by de Oliveira et al. (2020), we hypothesized that cows diagnosed with metritis and treated with CM would have reduced rumination and activity posttreatment than cows treated with ceftiofur and cows left untreated. In addition, we hypothesized that metritic cows would have decreased rumination and activity compared with nonmetritic (**NMET**) cows. Our objective was to characterize the rumination and activity of metritic cows treated with CM and ceftiofur (**CEF**) and cows left untreated (**CON**), using AHMD. A secondary objective was to compare behavioral patterns of metritic cows with NMET cows.

Cows used in this study were a subset of those enrolled in a larger experiment that evaluated the effect of different treatments on cure of metritis (de Oliveira et al., 2020). All animal procedures were approved by the University of Florida Institutional Animal Care and Use Committee (IACUC protocol numbers 201509189 and 201609403). At 220 ± 3 d of gestation, nulliparous Holstein cows (n = 311) were fitted with a collar containing an AHMD (HR-LDn tags, SCR Engineers Ltd.) that were placed on the left dorso-cranial area of the neck. The AHMD records raw rumination and activity data in real time, which is then processed by the DataFlowII (SCR Inc.), and averages were available for 2- and 24-h intervals. Daily averages were used in this study. The accuracy of rumination and activity recorded by AHMD in lactating dairy Holstein cows has been validated by Merenda et al. (2020), who observed high accuracy for rumination but low to high accuracy for different activity behaviors.

Cows were examined for diagnosis of metritis at 5, 7, and 9 DIM as previously described (de Oliveira et al., 2020). Cows with a fetid, watery, reddish-brownish discharge were diagnosed with metritis and the day of diagnosis was defined as d 0. At d 0, cows were randomly allocated to 1 of 3 treatments: CM (n = 45), intrauterine infusion of 24 g of CM dissolved in 40 mL of sterile distilled water at d 0, 2, and 4; CEF (n = 47), subcutaneous injection of 6.6 mg/kg of ceftiofur crystalline-free acid (Excede Sterile Suspension, Zoetis) on d 0 and 3; CON (n = 39), no treatment applied at metritis diagnosis. Although using a placebo is considered the gold standard for clinical trials, one was not used in this trial because of the difficulty in designing an appropriate one. Randomly selected NMET cows (n = 180) were matched to cows diagnosed with metritis according to age at calving and calving date. The mean (±SD) DIM at d 0 for CM, CEF, and CON was 6.9 ± 1.7, 7.0 ± 1.7, and 6.8 ± 1.5, respectively. Day 0 for NMET was 6.9 ± 1.7 DIM. This was a convenience sample; therefore, no a priori sample size calculation was performed.

On d 12, all cows in the CM, CEF, and CON treatments were re-examined to determine cure of metritis. Treatment success and cure occurred when the vaginal discharge on d 12 was mucoid and not fetid and cows did not receive antimicrobial escape therapy. All metritic cows were eligible to receive non-antimicrobial and antimicrobial escape therapy after d 0 if they exhibited signs of dehydration, anorexia, and weakness, or persistent rectal temperature ≥39.5°C. Antimicrobial escape therapy was determined by farm personnel and could include penicillin G procaine (Bactracillin G, Aspen), ampicillin trihydrate (Polyflex, Boehringer Ingelheim), or ceftiofur hydrochloride (Excenel, Zoetis).

Rumination and activity data, pre- (d −7 to −1) and post- (d 0 to 21) diagnosis were analyzed separately, excluding prepartum data. The association between treatment (CM vs. CEF vs. CON) and rumination and activity prediagnosis was analyzed by ANOVA for repeated measures using the MIXED procedure of SAS version 9.4 (SAS/STAT, SAS Institute Inc.), and no differences (*P* > 0.10) existed before treatment assignment. The models included the fixed effects of treatment, time (day relative to diagnosis), and the interaction between treatment and time. The effect of treatment on rumination and activity postdiagnosis was analyzed using similar models, but with the addition of rumination and activity data on d −1 as a covariate. When a main effect of treatment was observed, multiple comparisons were performed using Tukey adjustments. When an interaction between treatment and time was observed, multiple comparisons within day were performed using the SLICE option. The effect of treatment on the proportion of cows receiving escape therapy was analyzed by chi-squared test using the FREQ procedure of SAS. A separate analysis was performed to compare the treatments (CM vs. CEF vs. CON) with the NMET group as reference. When an effect of group was observed, multiple comparisons were performed using Dunnett adjustments, with the NMET group as reference. Statistical significance was defined as *P* ≤ 0.05 and statistical tendencies as 0.05 < *P* ≤ 0.10.

The percentage of cows diagnosed with metritis that were diagnosed as cured 12 d after enrollment were 47% (21/45), 70% (33/47), and 56% (22/39) for CM, CEF, and CON, respectively. Administration of escape therapy with any therapy was greater (*P* ≤ 0.05) for CM than CEF and CON (80.0 vs. 61.7 vs. 59.0%). Administration of escape therapy that included antimicrobials was greater for CM than CEF (22.2% vs. 6.4%; *P* = 0.03), but there was no difference between CM and CON (22.2 vs. 10.3%; *P* = 0.14). There was no difference (*P* = 0.59) in the interval from diagnosis to receiving antimicrobial escape therapy between treatments, and the mean (±SE) was 6.5 ± 0.4, 7.7 ± 0.3, and 5.8 ± 0.3 d for CM, CEF, and CON, respectively.

In Table 1 we present the results from the multivariable analyses of the effect of treatment (CM, CEF, and CON) on postdiagnosis rumination and activity. There was an effect of treatment (*P* < 0.01) on rumination and activity. The effect of treatment on rumination showed that cows in CM had (*P* < 0.01) lesser rumination (432 ± 11 min/d) than CEF (484 ± 10 min/d), and tended to have (*P* = 0.06) lesser rumination than CON (469 ± 12 min/d). There was no difference (*P* = 0.63) in rumination postdiagnosis between CEF and CON. There was an interaction (*P* < 0.05) between treatment and time on rumination postdiagnosis, and the interaction showed that CM had lesser (*P* < 0.05) rumination than CEF from d 1 to 11, d 18, and d 20; CM had lesser (*P* ≤ 0.05) rumination than CON from d 2 to 8, and CEF was not different from CON (Figure 1A). The effect of treatment on activity showed that cows in CM had (*P* < 0.05) lesser activity (628 ± 15 arbitrary units) than CON (684 ± 15 arbitrary units). There was no difference (*P* = 0.60) in activity postdiagnosis between CM and CEF. There was an interaction (*P* < 0.05) between treatment and time on activity, and the interaction showed that CM had lesser (*P* ≤ 0.05) activity than CON on d 2, from d 6 to 11, and d 13 to 14; CM was not different from CEF; and CEF had lesser (*P* ≤ 0.05) activity than CON on d 8, 9, 13, and 14 (Figure 1B).

**Table 1:** Results from a multivariable analysis postdiagnosis (0 to 21 d) evaluating the effect of treatments in rumination and activity^1^

Item	Treatment^2^		
	CM (n = 45)	CEF (n = 47)	CON (n = 39)
Rumination			
Postdiagnosis	432 ± 11a,b, A,B	484 ± 10a,b, A,B	469 ± 12a,b, A,B
Activity			
Postdiagnosis	628 ± 15^b^	648 ± 13^ab^	684 ± 15^a^

a,b Least squares means with different lowercase superscripts indicate a significant difference (*P* ≤ 0.05) between treatments.

A,B Least squares means with different uppercase superscripts indicate a significant tendency (0.05 < *P* ≤ 0.10) between treatments.

1 Values are LSM ± SEM of rumination and activity.

2 Primiparous Holstein cows diagnosed with metritis were randomly allocated to 1 of 3 treatments: CM (n = 45), intrauterine infusion of 24 g of chitosan microparticles on d 0, 2, and 4; CEF (n = 47), 6.6 mg/kg ceftiofur crystalline-free acid on d 0 and 3; and CON (n = 39), no treatment.

**Figure 1:**
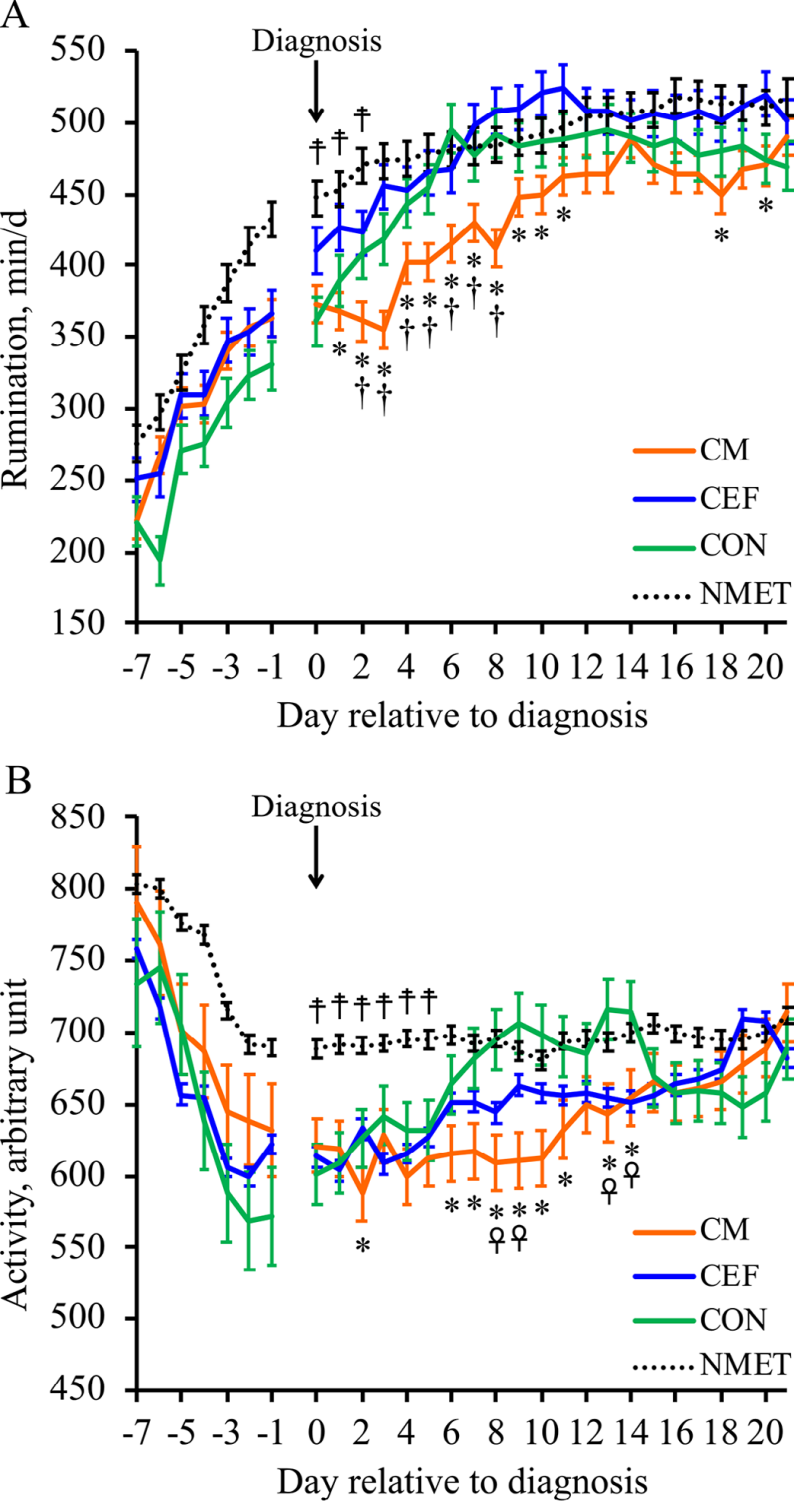
Association between metritis treatment or group and daily rumination (A) and daily activity (B). Day 0 = day of diagnosis. Primiparous Holstein cows diagnosed with metritis were randomly allocated to 1 of 3 treatments: CM (n = 45), intrauterine infusion of 24 g of chitosan microparticles on d 0, 2, and 4; CEF (n = 47), 6.6 mg/kg ceftiofur crystalline-free acid on d 0 and 3; and CON (n = 39), no treatment. NMET (n = 180) = cows not diagnosed with metritis. Within days relative to diagnosis, different symbols indicate that LSM between treatments differed (*P* ≤ 0.05; *CM vs. CEF, †CM vs. CON, ☥CEF vs. CON) or that CM, CEF, and CON differed from ☨NMET. Two analyses, one comparing treatments and another comparing treatments with NMET, were performed. Analysis 1: no effect of treatment on rumination (*P* = 0.12) or activity (*P* = 0.32) prediagnosis; effect of treatment *P* ≤ 0.03, time *P* ≤ 0.03, and treatment × time *P* ≤ 0.04 on rumination and activity postdiagnosis. Analysis 2: effect of group *P* < 0.01 and time *P* < 0.01, but not group × time *P* > 0.10 on rumination and activity prediagnosis; effect of group *P* < 0.01, time *P* < 0.01, and group × time *P* < 0.01 on rumination and activity postdiagnosis.

According to the model in which CM, CEF, CON, and NMET were included, there was an association between group (*P* < 0.01) and prediagnosis rumination and activity. Cows in CM, CEF, and CON had lesser (*P* ≤ 0.05) rumination and activity than NMET cows. Rumination for CM, CEF, CON, and NMET was 308 ± 14, 314 ± 13, 275 ± 15, and 356 ± 7 min/d. Activity for CM, CEF, CON, and NMET was 694 ± 21, 659 ± 19, 650 ± 22, and 749 ± 12 arbitrary units, respectively. Prediagnosis, there was no interaction between group and time (*P* > 0.10) on activity and rumination. Postdiagnosis, there was a main effect of group (*P* < 0.01) on rumination and activity. Cows in CM and CON had lesser (*P* ≤ 0.05) rumination than cows in NMET (492 ± 5 min/d), and NMET was not different from CEF. There was an interaction (*P* < 0.01) between groups and time on rumination postdiagnosis, and the interaction showed that CM had lesser (*P* ≤ 0.05) rumination than NMET from d 0 to 13, and from d 15 to 20; CEF had lesser (*P* ≤ 0.05) rumination than NMET from d 0 to 2, and d 10 to 11; and CON had lesser (*P* ≤ 0.05) rumination than NMET from d 0 to 4, and from d 17 to 21 (Figure 1A). Cows in CM and CEF had lesser (*P* ≤ 0.05) activity than cows in NMET (695 ± 8 arbitrary units), and NMET was not different (*P* = 0.34) from CON. There was an interaction (*P* < 0.01) between group and time on activity postdiagnosis, and the interaction showed that CM had lesser (*P* ≤ 0.05) activity than NMET from d 0 to 14; CEF had lesser (*P* ≤ 0.05) activity than NMET from d 0 to 5, and d 14 to 15, and CON had lesser (*P* ≤ 0.05) activity than NMET from d 0 to 5, and d 19 (Figure 1B).

In the current study, we compared the rumination and activity in metritic cows treated with CM or CEF with untreated cows using a subset of cows from a previous study (de Oliveira et al., 2020). As expected, we did not detect significant differences in rumination and activity prediagnosis. Postdiagnosis, we observed a major reduction in rumination in CM compared with CEF and CON, and also observed a reduction in activity in CM compared with CON, mostly from d 6 to 14.

Rumination is stimulated by mild tactile stimulation and low to moderate distension of the rumen by the ruminal contents on the tension receptors in the luminal surface of the reticulorumen (Ash and Kay, 1959). Therefore, rumination is affected by rumen fill and forage content of the diet. In the case of animals in the same diet, rumination is expected to be mainly affected by rumen fill. Indeed, over time there is a positive correlation between rumination time and DMI (Schirmann et al., 2012). Rumination can also be affected by inflammation (Borderas et al., 2008; Soriani et al., 2012). A reduction in rumination in cattle has been observed after LPS administration (Borderas et al., 2008). The onset of inflammation caused by LPS administration leads to the release of proinflammatory cytokines that can induce anorexia (Plata-Salamán et al., 1996) and lead to a reduction in rumen fill and rumination. In addition, the proinflammatory cascade can directly decrease rumination by acting though adrenergic receptors to induce gastric muscle relaxation and stasis (Leek, 2001). Therefore, the major decrease in rumination in CM may have been caused by an exacerbated inflammatory response, which may have caused a decrease in DMI, depression of the gastric centers causing stasis, or a combination of both. de Oliveira et al. (2020) demonstrated that treatment with CM resulted in greater culling due to inflammatory conditions such as metritis, pelvic inflammation, peritonitis, and mass in the pelvis than CEF or CON. Furthermore, treatment with CM resulted in decreased milk yield and increased culling for low milk yield, which may be a consequence of decreased DMI. Additionally, a greater proportion of cows in CM required antimicrobial escape therapy (de Oliveira et al., 2020), which was applied when the condition did not improve or worsened. Therefore, it is reasonable to speculate that CM administered into an inflamed uterus might further exacerbate the inflammatory process. Indeed, an increase in leukocyte infiltration into the mammary gland (Lanctôt et al., 2017) and lungs (Huang et al., 2005) has been observed after administration of chitosan.

Interestingly, cows in CEF had similar rumination postdiagnosis compared with CON, despite CEF having been reported to have greater cure rate (McLaughlin et al., 2012; de Oliveira et al., 2020) and increased milk yield compared with CON (Piccardi et al., 2016; de Oliveira et al., 2020). It is not clear why the improvement in cure rate and milk yield was not accompanied by greater rumination herein. It is possible that the administration of escape therapy to cows that did not improve or worsened after metritis diagnosis was sufficient to ameliorate rumination in the CON group. To our knowledge, this is the first study to describe the pattern of rumination in untreated metritic cows; therefore, additional experiments are needed to improve our understanding of withholding antimicrobial treatment at the time of metritis diagnosis on rumination.

Similar to the results for rumination, CM had decreased activity compared with CON. Interestingly, CON had a faster recovery in activity than CEF, despite the reported increase in cure rate and milk yield for CEF compared with CON (de Oliveira et al., 2020). We explored the difference in activity between CEF and CON by looking at the percentage of cows receiving antimicrobial escape therapy, which would require a move to the hospital pen. Pen changes are expected to result in increased activity (Hasegawa et al., 1997). However, the proportion of cows receiving escape therapy was similar between CON and CEF.

Our results also revealed that cows diagnosed with metritis had decreased rumination and activity compared with cows not diagnosed with metritis, particularly before diagnosis. Similar to our findings, others have also observed reduced rumination and activity before and after metritis diagnosis (Liboreiro et al., 2015; Stangaferro et al., 2016; Merenda et al., 2021b).

In conclusion, CM used as described herein decreased rumination and activity compared with CON, which indicates a negative systemic effect of CM. This corroborates and complements our previous observations in the larger study evaluating cure and performance. Additionally, metritic cows had decreased rumination and activity prediagnosis, which may allow for the use of AHMD for diagnosing metritis.
